# Respiratory syncytial virus (RSV) enhances translation of virus-resembling AU-rich host transcripts

**DOI:** 10.1186/s12985-025-02838-z

**Published:** 2025-07-15

**Authors:** Kyra Kerkhofs, Nicholas R. Guydosh, Mark A. Bayfield

**Affiliations:** 1 Department of Biology, Faculty of Science, York University, Toronto, ON M3J 1P3 Canada; 2 Section On mRNA Regulation and Translation, Laboratory of Biochemistry & Genetics, National Institute of Diabetes and Digestive and Kidney Diseases, National Institutes of Health, Bethesda, MD 20892 USA

**Keywords:** RSV, VSV, Translation efficiency, Polysome profiling, High-throughput sequencing, AU-rich transcripts

## Abstract

**Background:**

Viruses strongly rely on the host’s translational machinery to produce viral proteins required for replication. However, it is unknown how viruses that do not globally inhibit cap-dependent translation compete with abundant host transcripts for ribosomes. Viral infection often triggers eukaryotic initiator factor 2α (eIF2α) phosphorylation, leading to global 5’-cap-dependent translation inhibition. Respiratory syncytial virus (RSV) encodes mRNAs mimicking 5’-cap structures of host mRNAs and thus inhibition of cap-dependent translation initiation would likely also reduce viral translation.

**Methods:**

RSV-infected HEp-2 and A549 cells were analyzed to determine translation levels using western blotting, indirect immunofluorescent staining and polysome profiling. Transcriptome-wide translation efficiencies of virus-infected cells were compared against mock-infected cells using high-throughput sequencing of poly(A)-tail enriched total mRNA and transcripts associated with heavy polysomes.

**Results:**

We confirmed that RSV limits widespread translation initiation inhibition and unexpectedly found that the fraction of ribosomes within polysomes increases during infection, indicating higher ribosome loading on mRNAs during infection. High-throughput sequencing revealed that virus-resembling, AU-rich host transcripts become more efficient at ribosome recruitment. Using a previously published dataset, we observe similar trends in another negative-sense single-stranded RNA virus, vesicular stomatitis virus (VSV).

**Conclusions:**

These findings revealed that RSV changes the translational landscape by enhancing translation of virus-resembling AU-rich host transcripts rather than inhibiting host translation.

**Supplementary Information:**

The online version contains supplementary material available at 10.1186/s12985-025-02838-z.

## Background

Viral infection often results in remodeling of the host’s translational landscape due to viral proteins that hijack translation regulatory factors, high numbers of viral transcripts and host-induced innate immune activation. Viruses rely completely on the host’s ribosomes for viral protein production and thus compete with host mRNAs [1]. Cells exposed to stress often regulate gene expression through 5’-cap-dependent translation arrest, often mediated through phosphorylation of the α-subunit of eIF2 (eIF2α) by stress-activated kinases, leading to inhibition of subsequent rounds of initiation. Without translation initiation, ribosome-free transcripts are bound by RNA-binding proteins and assemble into stress granules [2, 3].

Respiratory syncytial virus (RSV) is an enveloped virus containing a non-segmented, single-stranded, negative-sense RNA genome expressing 10 individually 5’-capped and polyadenylated transcripts transcribed by the viral polymerase [4–7]. Following fusion of the viral particle with the host’s membrane, the nucleocapsid is released into the cytoplasm and the viral polymerase (containing phosphoprotein RSV-P and large polymerase protein RSV-L) starts replication and transcription of the viral genome [8, 9] within cytoplasmic membraneless inclusion bodies [10–12]. Transcription of RSV transcripts requires an additional protein, RSV-M2-1, which functions as a transcription processivity factor [13, 14]. Viral mRNAs are transcribed in a sequential manner, starting from the 3’-end of the non-segmented negative-sense genome towards the 5’-end, leading to a gradient in expression as the viral polymerase undergoes a start-stop mechanism and reinitiates transcription at each gene [15]. M2-1 has also been shown to bind nascently transcribed viral transcripts and transport these from inclusion bodies into the cytoplasm [12, 16, 17]. Translation of the viral transcripts occurs in the cytoplasm using the host’s ribosomes [12, 18].

Since RSV transcripts mimic post-transcriptional features of host transcripts [19], it would be detrimental to viral gene expression if 5’-cap-dependent translation initiation were inhibited through eIF2α phosphorylation by stress-activated kinases. During viral infection, the integrated stress response is typically induced after detection of viral double-stranded RNA by the kinase PKR. RSV infection results in both upregulation of the stress-activated kinase PKR [20–22] and PKR activation through dimerization and autophosphorylation [23, 24]. This normally induces eIF2α phosphorylation leading to reduced translation initiation and stress granules formation. Multiple studies have demonstrated that RSV has developed different strategies to maintain host translation levels by negating eIF2α phosphorylation [21, 25–27]. This is achieved through direct binding of RSV-N to PKR and thereby limiting accessibility to eIF2α [21]. As a result, eIF2α phosphorylation is avoided despite PKR activation. However, another study reports that RSV induces stress granules [28]. Despite elucidation of inhibitory eIF2α phosphorylation strategies, stress granule formation during RSV infection remains controversial. Additionally, since RSV does not induce a strong “host shutoff” by inhibiting global 5’-cap-dependent translation initiation [21, 29], it remains to be determined how RSV successfully competes with host transcripts for the machinery required for translation of its viral genes.

In this study, we describe host translatome changes during infection that include preferential translation of transcripts more similar to viral transcripts. We first confirm that RSV limits inhibition of widespread translation initiation by lack of both eIF2α phosphorylation and stress granule formation. Interestingly, we found that the number of ribosomes within polysomes increases during infection, indicating enhanced ribosome loading. Next, through high-throughput sequencing of total and polysome-associated transcripts, we describe how transcripts that are normally lowly translated become more efficient at recruiting ribosomes during infection. Lastly, we show that more efficiently translated host transcripts are AU-rich, similar to viral transcripts.

## Methods

### Cell culture, RSV infection and arsenite treatment

HEp-2 cells (a HeLa-derived epithelial cell line) were grown in DMEM containing 5% FBS. A549 cells (a lung epithelial cell line) were grown in DMEM containing 10% FBS. Cell lines were maintained in a humidified incubator at 37 °C with 5% CO_2_. Arsenite (NaAsO_2_) treatment was done by incubating cells with 0.5 mM (unless otherwise stated) for 1 h at 37°C.

The RSV strain A2 (ATCC, serial passage-2 (P2)) was propagated in HEp-2 cells. Briefly, 15-cm plates at 80% confluency were infected with RSV (P2, MOI = 0.1) for 2 h at 37 °C in 5 mL FBS-free DMEM. Following infection, the cells were maintained in DMEM containing 1% FBS and incubated for approximately 3 days until syncytia formed. The cells were scraped, and supernatant was collected following centrifugation at 300 g for 15 min at 4°C. The RSV stock P3 was aliquoted, snap frozen in liquid nitrogen and stored at −80°C. Titration of the RSV stock was performed according to the Tissue Culture Infectious Dose-50 (TCID_50_) Spearman–Kärber method [30]. RSV P3 stock titer was confirmed through indirect immunofluorescent staining using polyclonal anti-RSV antibody (Virostat cat# 6010, data not shown).

For experiments, RSV infections were done using the titrated RSV P3 stock. In brief, cells were grown overnight and the RSV P3 stock, quickly thawed at 37 °C and diluted in FBS-free DMEM to the desired MOI. Generally, an MOI of 2.5 was used to infect more than 75% of the monolayer, unless stated otherwise. The cells were washed once with PBS, followed by incubation with a small volume of FBS-free DMEM (*i.e.* 15 cm plates: 5 mL, 10 cm plates: 2 mL, 24-well plates: 200 μL, 96-well plate: 32 μL) and incubated for 2 h with frequent rocking to redistribute the infection medium evenly. Mock treatment included PBS wash and 2 h incubation in FBS-free DMEM. Following infection, the cells were maintained in DMEM containing 5% (HEp-2) or 10% inactivated FBS (A549) (30 min at 56 °C) for 24 h unless stated otherwise.

### Polysome profiling

**Fig. 1:**
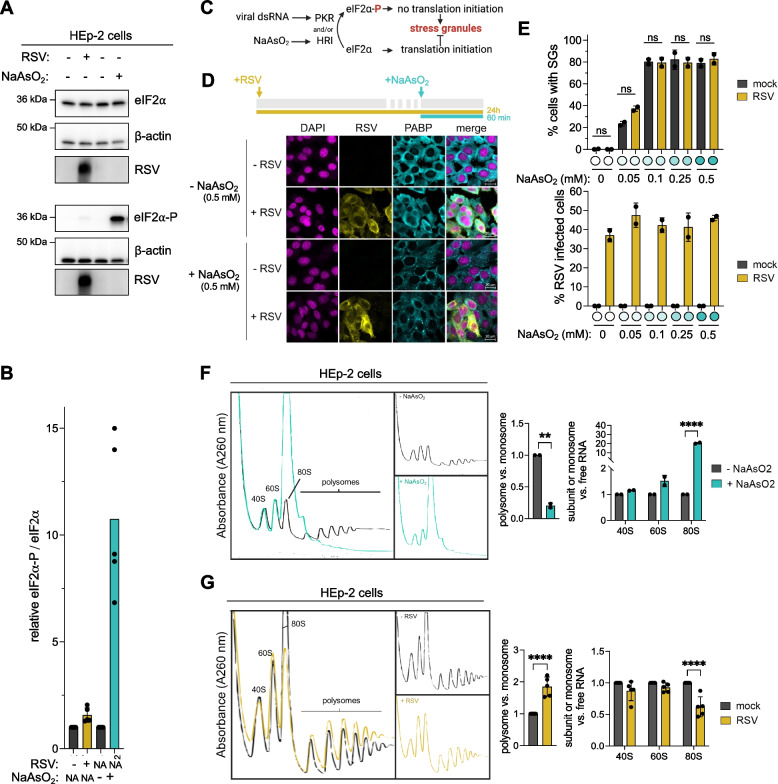
RSV infection maintains translation and increases polysome occupancy. **A** RSV infection only induces low levels of eIF2α phosphorylation. Western blot comparing eIF2α-P and total eIF2α levels between mock- and RSV-infected (MOI 2.5, 24 h) and untreated and NaAsO_2_-treated (positive control) (0.5 mM, 1 h) HEp-2 cells. RSV infection was confirmed by immunoblotting with a polyclonal anti-RSV antibody (pAb). β-actin serves as a loading control. **B** Relative quantification between eIF2α-P and total eIF2α levels against control cells (*n* = 5). P values were calculated with one-way ANOVA with Sidak’s multiple comparisons test. **C** Schematic representation of eIF2α-phosphorylating kinases activated during NaAsO_2_ stress (HRI) and viral infection (PKR). **D** RSV does not inhibit NaAsO_2_-induced stress granule formation. Indirect immunofluorescent staining of mock- and RSV-infected (MOI 2.5, 24 h) and NaAsO_2_-treated (0.5 mM, 1 h) HEp-2 cells (*n* = 1). DAPI staining identifies nuclei, PABP detects stress granules and RSV infected cells were detected with a polyclonal RSV antibody. **E** Quantification of mock- and RSV-infected HEp-2 cells (MOI 1.25, 24 h) treated with different concentrations of NaAsO_2_ (1 h). More than 200 cells were quantified at 20X magnification (*n* = 2). Number of cells were determined by DAPI, stress granules by PABP and RSV infection by polyclonal RSV staining. P values were calculated with one-way ANOVA with Tukey’s multiple comparisons test. **F,G** RSV redistributes 80S monosomes to polysomes. Polysome profiles of (**F**) untreated and NaAsO_2_-treated (0.5 mM, 1 h) and (**G**) mock- and RSV-infected (MOI 2.5, 24 h) sucrose gradient fractionated HEp-2 cells. AUC quantification between polysomes and monosomes (40S, 60S and 80S) are plotted to estimate translation levels. AUC quantification between free RNA fraction (not shown) and 40S, 60S and 80S are plotted to determine changes in free monosomes and 80S subunits. *P* values were calculated with an unpaired t-test for polysome vs monosome comparisons and a two-way ANOVA with Sidak’s multiple comparisons test. AUC: area under the curve

To determine translation levels, polysome profiling was performed as described in [31]. In brief, 100 μg/mL cycloheximide was added to the cells for 5 min at 37 °C prior to collection. The cells were washed twice in PBS containing 100 μg/mL cycloheximide. The cell pellets were stored at −80°C until use. Next, the cell pellets were lysed in 485 μL hypotonic buffer [5 mM Tris–HCl (pH 7.5), 2.5 mM MgCl2, 1.5 mM KCl, 1X Halt™ Protease Inhibitor Cocktail, 100 μg/mL cycloheximide, 2 mM DTT, 200 Units/mL SUPERase In™ RNase Inhibitor, 0.5% (v/v) Triton X-100 and 0.5% (w/v) sodium deoxycholate], followed by centrifugation for 5 min at 20,000 g at 4 °C to obtain cytoplasmic extracts. A fraction of the lysate was taken as the input sample. The remaining sample (500 μL of 20 A260 units) was fractionated on a 7-step 20–50% sucrose gradient prepared in sucrose buffer [20 mM HEPES (pH 7.6), 100 mM KCl, 5 mM MgCl2 and 100 μg/mL cycloheximide] by centrifugation at 30,000 RPM for 3 h in a Beckman SW41Ti rotor at 4 °C (acceleration: max., deceleration: no brake). Polysome profiles were obtained with BRANDEL Density Gradient Fractionation System by measuring the absorbance at 254 nm with the UA-6 Detector in a continuous flow. Polysome fractions were collected (800 μL each). Polysome traces were obtained with the build-in chart recorder with paper and pen and digitally represented using Inkscape v.1.2.1. Sensitivity of chart recorder for recording polysomes traces was used at sensitivity of 2 (NaAsO_2_ samples) and sensitivity of 1 (RSV samples). P values were calculated with an unpaired t-test for polysome vs monosome comparisons and a two-way ANOVA with Sidak’s multiple comparisons test (Fig. 1F, G and Supplementary Fig. S1G).

RNA extraction of polysome fractions was performed by adding 2 parts 100% ethanol containing 80 mM NaOAc, pH 5.1 and 300 μg GlycoBlue overnight at −80°C to precipitate the RNA. RNA pellets were collected by centrifugation at 20,000 g for 30 min at 4 °C, followed a 70% ethanol wash and resuspension in ddH_2_O. Both polysomal RNA and total RNA were extracted using Trizol according to manufacturer’s instruction.

### Western blot and protein quantification

Cellular lysates were quantified using the Pierce Coomassie Plus (Bradford) Assay Reagent to obtain protein concentrations. Protein samples were incubated with 1 × Laemmli buffer [5X Laemmli buffer: 5% β-mercaptoethanol (v/v), 0.02% bromophenol blue (w/v), 30% glycerol (v/v), 10% sodium dodecyl sulfate (SDS) (w/v), 250 mM Tris–HCl, pH 6.8] for 10 min at 95 ºC and separated using a 12% SDS-PAGE for 1 h at 110 V or a 4–20% gradient Mini-PROTEAN Tris–HCl gel (BioRad) for 35 min at 200 V. Proteins were transferred to a nitrocellulose membrane for 2 h at 50 V or a PVDF membrane using the Trans-Blot Turbo System (1.3 A, 7 min). The membrane was blocked in 5% nonfat dried milk (NFDM) in tris-buffered saline containing 0.1% Tween-20 (TBS-T) for 1 h at room temperature or overnight at 4ºC. The membrane was probed with appropriate primary antibodies in TBS-T for 1 h or overnight at room temperature. After primary antibody binding, the membrane was washed 5 times in TBS-T, incubated with appropriate HRP-coupled secondary antibody for 1 h at room temperature and washed 5 times in TBS-T. Membranes were incubated with Pierce® ECL Western Blotting Substrate or Clarity Western ECL substrate and imaged on a MicroChemi chemiluminescence system (DNR Bio-Imaging Systems) or a Amersham Imager 600. Primary antibodies used were goat anti-RSV (Virostat 0601), rabbit anti-eIF2α S51-P (Abcam ab32157), mouse anti-eIF2α (Cell Signaling Technology L57A5), rabbit anti-eIF2α (Bethyl A300-721A), mouse β-actin (Abcam ab8224), mouse anti-GAPDH (SantaCruz Biotechnology sc47724), rabbit anti-GAPDH (Abcam ab9485), rabbit anti-PKR (Abcam ab32506), mouse anti-RSV-M2-1 (Abcam ab94805), mouse anti-RSV-P (Abcam ab94965), and mouse anti-RSV-N (Abcam ab94806). Secondary antibodies used were anti-mouse IgG HRP (1:10,000), anti-rabbit IgG HRP (1:10,000) and anti-Goat IgG HRP (1:10,000). Western blot quantifications were done using Image J v2.14.0.

In order to probe the same membrane for proteins with similar molecular weight with multiple primary antibodies raised in different species (*e.g.* rabbit anti eIF2α-P and mouse anti-eIF2α) we performed mild stripping of the western blot membranes to quench HRP. In brief, the membrane was incubated twice in stripping buffer-HCl, pH 2.2 [1.5% glycine (w/v), 0.1% SDS, 1% Tween-20] for 10 min, followed by two 5-min washes with PBS and two 5 min washed with TBS-T. Prior to probing with primary antibody, the membrane was blocked as described above.

Quantification of the ratio between phosphorylated and total eIF2α was done by taking a direct ratio between these proteins (*i.e.* rabbit anti-eIF2α-P/mouse anti-eIF2α). In case of rabbit anti-eIF2α-P and rabbit anti-eIF2α quantifications, separate membranes were prepared and both probed with β-actin loading control. Quantification of the ratio between phosphorylated and total eIF2α was done by taking β-actin into account (*i.e.* [eIF2α-P/β-actin]/[eIF2α/β-actin]).

### Indirect immunofluorescent staining

The intracellular localization of endogenous expressed proteins was determined through indirect immunofluorescent staining. Prior to fixing, monolayers were washed twice with PBS. Next, cells were fixed for 20 min with 4% paraformaldehyde in PBS and permeabilized for 10 min with 0.1% Triton X-100 in PBS. Next, the cells were blocked for 1 h in 1% Bovine Serum Albumin (BSA) in PBS, followed by incubation with primary antibodies in blocking solution for 1 h at room temperature or overnight at 4ºC. Cells were subsequently washed 4 times with PBS, incubated with appropriate fluorochrome-bound secondary antibodies for 1 h at room temperature and washed 4 times with PBS. Cells were stained for 2 min with 2.5 μg/mL 4',6-diamidino-2-phenylindole (DAPI), washed twice with PBS and overlaid with 1,4-diazabicyclo[2.2.2]octane (DABCO). All images were acquired by the LSM700 laser scanning confocal microscope (Zeiss) with a 63 × oil immersion objective or 20 × objective.

Primary antibodies used were rabbit anti-PABP (Abcam ab21060), mouse anti-G3BP (BD Biosciences, 611126), goat anti-RSV (Virostat, 0601). Secondary antibodies used were donkey anti-rabbit IgG Alexa Fluor 594 (1:1000), donkey anti-mouse IgG Alexa Fluor 546 (1:1000), and donkey anti-goat IgG Alexa Fluor 488 (1:1000). Immunofluorescent images were pseudo-colored: (1) blue-emitting fluorescent DAPI to magenta, (2) RSV-specific staining (green-emitting) to yellow and (3) host proteins (red-emitting) to cyan. *P* values between mock- and RSV-infected cells at different NaAsO_2_ concentrations (Fig. 1E) were calculated with one-way ANOVA with Tukey’s multiple comparisons test.

### RT-qPCR

RNA for total and polysomal fractions was extracted using Trizol extraction (according to manufacturer's instructions) from non-targeting shRNA expressing HEp2 cells. The isolated RNA was resuspended in 1X Reaction Buffer with 2 units of Turbo DNase (ThermoFisher Scientific) and incubated for 30 min at 37°C. The DNase-treated samples were subsequently Trizol extracted again to inactivate DNase activity. Next, 50 ng/μL DNase-treated RNA was reverse transcribed using the iScript cDNA Synthesis Kit (Bio-Rad) according to manufacturer′s instructions. The cDNA was diluted to 25 ng (in 12.5 μL per technical triplicate) and quantified using the SensiFAST SYBR No-Rox kit (Meridian Bioscience) with 12.5 μM of each primer (1 × forward, 1 × reverse) (Supplementary Table S9) using following qPCR settings: 95 °C for 5 min and 35 cycles of 5 s at 95 °C and 15 s at 60 °C, followed by a melting curve analysis up to 99 °C to confirm amplification of a single amplicon. Fold enrichment was calculated using the ΔCt method (Ct _5.8S rRNA_ – Ct _RNA_). Translation efficiency (TE; polysomal RNA/input RNA) fold enrichment for TE RSV/TE mock was calculated by the ratios of ΔΔCt.

### Next generation sequencing, mapping and sample quality control

#### Sample quality control

Following Trizol extraction of total and polysomal RNA from non-targeting shRNA expressing HEp2 cells (see polysome profiling methods section), 5 μg RNA was heated in 1X formamide [2X formamide: 95% deionized formamide, 0.025% (w/v) bromophenol blue, 0.025% xylene cyanol (w/v), 5 mM EDTA, pH 8.0] for 3 min at 95 °C, and immediately snap cooled on ice for 3 min. Next, the RNA was separated on a 1% agarose gel in 1X tris–borate-EDTA (TBE) buffer for 40 min at 100 V to determine the RNA quality before library preparation. Bioanalyzer data quality control of RNA samples performed at TCAG, Hospital for Sick Children, showed an RNA integrity number (RIN) of 10 for all samples.

#### Next generation sequencing and mapping of RSV-infected sample reads

RNA extracted from total and polysomal RNA from non-targeting shRNA expressing HEp2 cells (see polysome profiling methods section) was subjected to stranded cDNA library preparation by poly(A) tail selection (poly(A) +) (NEBNext) and paired end 50 bp sequencing using the Illumina NovaSeq 6000 at The Centre for Applied Genomics (TCAG, Hospital for Sick Children). Quality of the raw reads in fastq format was verified using FastQC 0.12.1. The raw reads were aligned to the concatenated GRCh38 GENCODE release 39, and RSV genome (GenBank: KT992094.1) using STAR aligner v.2.7.11b [32]. Prior to mapping, the concatenated genome was indexed using STAR v.2.7.11b with settings *–runMode genomeGenerate –sjdbOverhang 99.* Gene expression analysis was done using the function featureCounts of package subread v.2.0.6 (setting: *-t exon*) to obtain raw counts (Supplementary Table S1). Bigwig files for IGV viewing were prepared using samtools’ bamCoverage with settings *-bs 1 –normalizeUsing BPM*.

#### Mapping of VSV-infected sample reads

Raw reads in fastq format for the VSV dataset were downloaded from Sequence Read Archive (SRP158625) and analyzed similarly as above with mapping of the raw reads against the indexed concatenated GRCh38 GENCODE release 39 and VSV genome (GenBank: OR921183.1) using STAR aligner 2.7.11b [32]. Raw counts were obtained using the function featureCounts of package subread v.2.0.6 (setting: *-t exon*).

### Differential expression analysis

Raw counts were used for differential expression analysis using DESeq2 v.1.32.0 to obtain DESeq2-normalized counts through the median of ratios method (*i.e.* normalized for sequencing depth and RNA composition) (Supplementary Tables S2, S3) [33]. The DESeq2 design matrix contained information for the component *virus* (distinguishing between mock- and RSV-infection) and *RNA* (distinguishing between poly(A)-enriched total RNA and poly(A)-enriched polysomal RNA). Differential expression analysis comparing total and polysomal RNA between mock- and RSV-infected used the design formula *design* = ~ *virus* (Supplementary Tables S2, S3). While differential expression analysis comparing translation efficiency (TE; polysomal read counts/total read counts) between mock- and RSV-infected used the design formula *design* = ~ *RNA* + *virus* + *RNA:virus* which takes the TE ratio into consideration (Supplementary Table S4). Reproducibility between biological replicates was determined through multidimensional scaling (MDS) using R package EdgeR v.4.4.2 after *log2* + *1* transformation of the reads (Supplementary Fig. S2C). In addition, reproducibility between replicates was determined through calculating the Euclidean distance of the gene expression matrix from different samples plotted on a heatmap using R package pheatmap v.1.0.12 (Supplementary Fig. S2D).

**Fig. 2:**
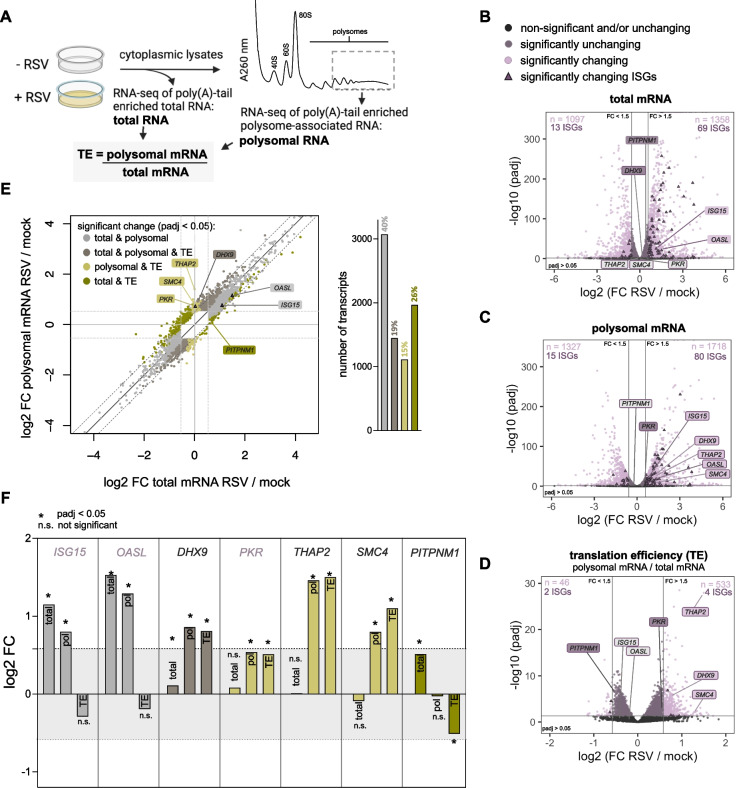
RSV infection induces distinct modes of host translation changes. **A** Schematic representation of experimental design. Cells were mock- (- RSV) or RSV-infected (+ RSV) with a multiplicity of infection (MOI) of 2.5 for 24 h. Prior to harvest, cells were treated with cycloheximide (CHX) to halt translation elongation and stabilize ribosomes on mRNA. Cell lysates were fractionated on sucrose gradients separating 40S, 60S, 80S and polysomes. RNA was isolated from heavy polysomes and from total RNA (acquired prior to fractionation), poly(A)-tail enriched and analyzed by next-generation sequencing. Differential expression analysis was completed by DESeq2. Translation efficiency (TE) was calculated by DESeq2 by taking ratios between polysomal and total RNA. **B-D** Volcano plots reveal differentially expressed host mRNAs between mock- and RSV-infected samples (MOI 2.5, 24 h) (three biological replicates) from total mRNA (**B**), polysomal mRNA (**C**) and translation efficiency (the ratio between polysomal and total mRNA) (**D**). Fold change (FC) and adjusted *P* values (padj) were obtained by DESeq2. The horizontal line indicates a cutoff of padj < 0.05 and vertical lines indicate a 1.5-FC. Significantly up- or downregulated transcripts are highlighted in light purple. Significantly up- or downregulated interferon stimulated genes (ISGs) are shown as dark purple triangles. The number of up- and downregulated transcripts are shown within each plot. Examples transcripts are shown on each plot with their label color corresponding to their significance and fold change. **E** Correlation analysis of changes between total mRNAs (RSV/mock) and polysome associated mRNAs (RSV/mock) with individual transcripts from volcano plots in **B**-**D** highlighted (*left*). Horizontal and vertical lines indicate a 1.5-FC. Diagonal lines indicate boundaries outside of which transcripts have significantly changing translation efficiencies (TE). Transcripts are color-coded based on significant (padj < 0.05) changes in total, polysomal and TE. Light grey: significant change in total and polysomal mRNA, without TE changes. Dark grey: significant change in total mRNA and stronger change in polysomal mRNA, leading to TE changes. Light green: significant change in polysomal mRNA, without changes in total mRNA, leading to changes in TE. Dark green: significant change in TE counteracting changes in total RNA. Bar plot of percentage of significantly (padj < 0.05) changing transcripts using the same color scheme (*right*). FC: fold change. **F** Examples of individual transcripts displayed throughout **B**-**E**. Bar graphs of transcripts are color-coded as in **E,** based on significant changes in total, polysomal and TE between mock- and RSV-infected cells. ISGs are shown in purple. The lines surrounding the light grey box indicate fold changes outside the 1.5-fold change cutoff

**Fig. 3:**
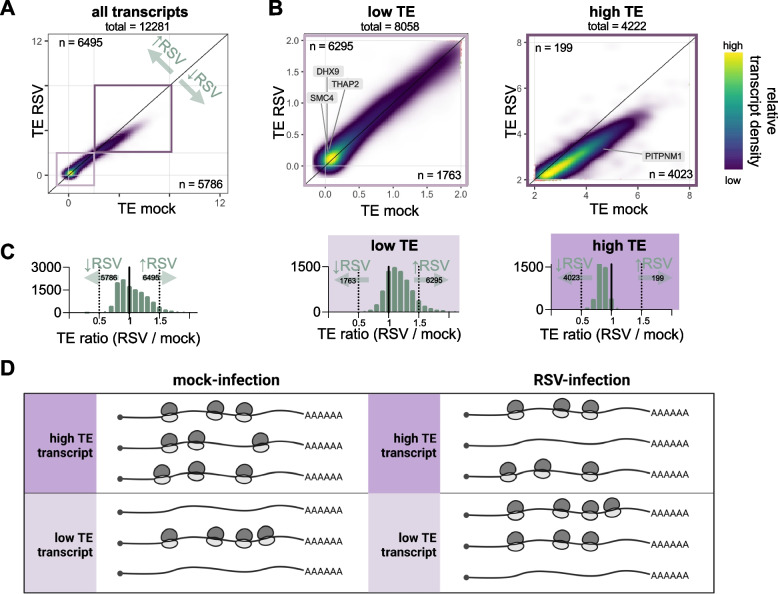
Transcripts with low TE become more efficient at ribosome recruitment during RSV infection. **A,B** Density scatterplots of TE between mock- and RSV-infected samples with a global overview (**A**) and zoomed versions (**B**). These plots demonstrate that RSV infection tends to reduce the TE of high TE transcripts and increase the TE of low TE transcripts. The color gradient represents the density of the dots with most abundant region shown in yellow and least abundant region shown in dark purple. The transcript density color gradient is relative and rescales in each individual plot. Transcript counts computed for any gene with TE change during infection, regardless of level of significance. The light purple box indicates low TE transcripts while the dark purple box indicates high TE transcripts. The number of transcripts on each side of the diagonal is displayed. **C** Histograms corresponding to scatterplots in **A** and **B** are shown below each graph representing the fold-change between TE for mock- and RSV-infected samples, to further illustrate how RSV infection differentially affects transcripts that are low- and high-TE under mock-infected conditions. **D** Schematic representation summarizing results from **A**-**C**. Transcripts are represented as heavily loaded with ribosomes or as non-translating mRNA since the experimental design was to measure heavy polysome-associated mRNA or input RNA for TE calculations. Some transcripts that are heavily loaded with ribosomes in the mock-infected condition (high TE) lose ribosomes after RSV infection. Conversely, some transcripts that are not loaded with ribosomes in the mock-infected condition (low TE) are loaded with ribosomes during RSV infection

**Fig. 4:**
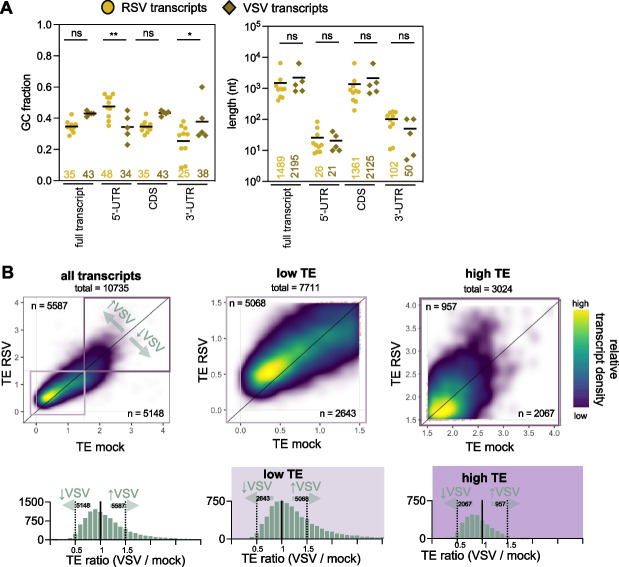
VSV infection induces the same relative enhanced ribosome recruitment for transcripts with low TE. **A** Distribution of GC% and length of viral protein-coding transcripts comparing RSV and VSV. Average GC% and length values are displayed underneath and shown as horizontal lines. P values were calculated with one-way ANOVA with Sidak’s multiple comparisons test (*P* values: * < 0.05, ** < 0.01, ns: not significant). This analysis shows that the transcriptomes of the viruses are similar. **B** Translation efficiency (TE) calculated from normalized reads as in Fig. 3A [34]. Density scatterplots of TE compare mock- and VSV-infected samples (MOI 10, 6 h) with a global overview (*top)* and corresponding histograms *(bottom)* shown representing the fold-change between mock- and VSV-infected samples for all, low TE (< 1.5) and high TE (> 1.5) transcripts. This analysis reveals that VSV globally changes TE in a way that is similar to RSV

Volcano plots (Fig. 2B-D), scatterplots displaying log2FC (RSV/mock) (Fig. 2E) and density scatterplots displaying TE (Figs. 3 A, B and 4B) were generated using R package ggplot2 v.3.5.1 using protein coding transcripts only, as defined in.gtf (hg38 refGene). Histograms (Figs. 3C and 4B), cumulative histograms (Supplementary Fig. S4A) and bar graphs (Fig. 2E, F, Supplementary Fig. S3B and Supplementary Figs. S5B, C) were generated in GraphPad Prism v.10.2.2.

### GO analysis

GO analysis was completed for differentially upregulated transcripts (Supplementary Table S5) using ShinyGO 0.76 (FDR cutoff 0.05; pathway size min 2; max 2000) [35]. The fold enrichment, enrichment false discovery rate (FDR) and number of genes in each cohort for GO biological processes of upregulated total mRNAs, polysomal mRNAs and TEs during RSV infection were obtained and displayed using GraphPad Prism v.10.2.2.

### 5’-UTR, CDS and 3’-UTR analysis

**Fig. 5:**
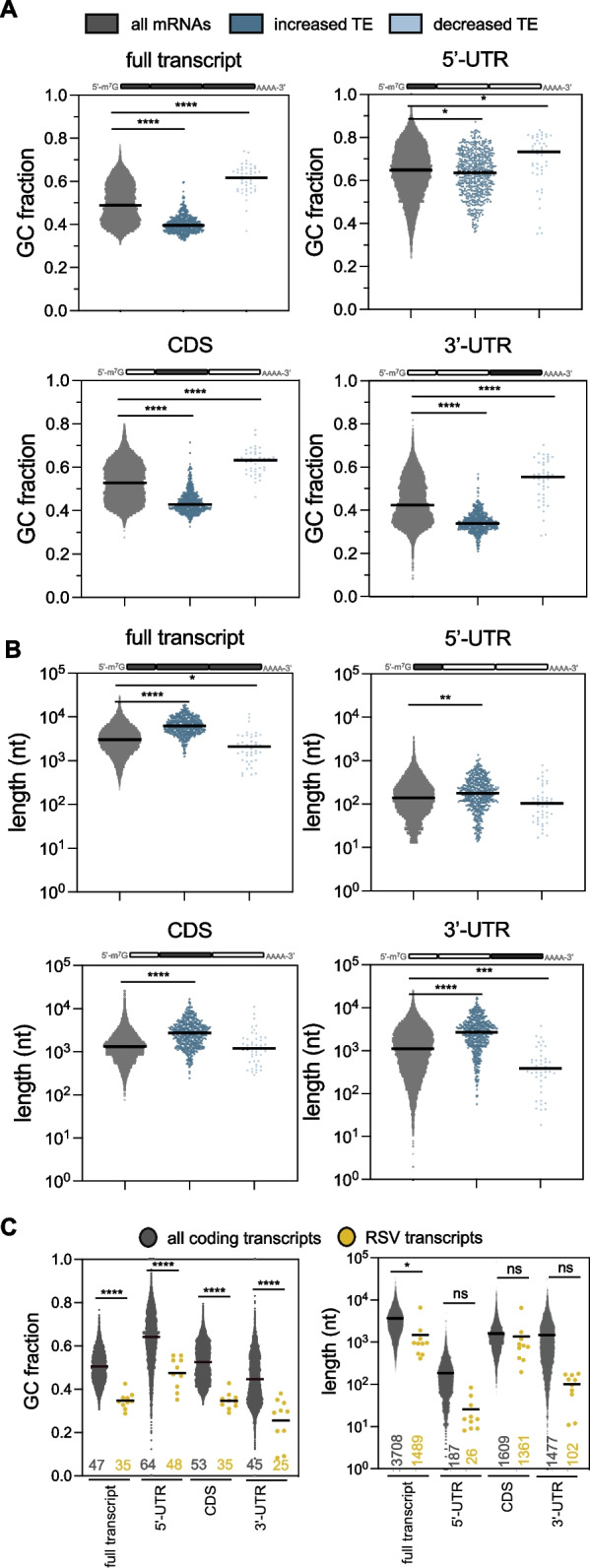
Transcripts with increased TE during RSV infection are more AU-rich and contain longer CDSs and 3’-UTRs. **A,B** Distributions of GC-content (**A**) and transcript length (**B**) of host protein-coding transcripts with significantly increased or decreased abundance and TE comparing RSV- and mock-infected samples (FDR < 0.05, FC > 1.5 and FC < 1.5). *P* values were calculated with one-way ANOVA with Tukey’s multiple comparisons test (*P* values: **** < 0.0001, *** < 0.001, ** < 0.01, * < 0.05). Averages are shown as horizontal lines. **C** Distributions of GC-content and length of viral protein-coding transcripts compared to all host mRNAs. Average GC% and length values are displayed underneath and shown as horizontal lines. P values were calculated with one-way ANOVA with Sidak’s multiple comparisons test (*P* values: **** < 0.0001, * < 0.05, ns: not significant)

Transcript sequences were downloaded from the Matched Annotation from the NCBI and EMBL-EBI (MANE) transcriptome (MANE.v1.3 https://ftp.ncbi.nlm.nih.gov/refseq/MANE/MANE_human/release_1.3/MANE.GRCh38.v1.3.refseq_rna.fna.gz), to obtain information for representative transcripts within the human transcriptome [36]. The RSV genome was downloaded from NCBI (GenBank: KT992094.1, https://www.ncbi.nlm.nih.gov/nuccore/KT992094.1) and the VSV genome from NCBI (GenBank: OR921183_1, https://www.ncbi.nlm.nih.gov/nuccore/2635771998). GC% and length was calculated for the MANE transcriptome based on CDS annotations found in the gbff file using a custom Python script. GC% and length of viral transcripts was calculated using a custom Python script (Supplementary Table S8). P values comparing GC% and length between low and high TE transcripts were calculated with an unpaired t test (Supplementary Fig. S3A and Supplementary Fig. S4B). P values comparing GC% and length between RSV and VSV transcripts were calculated with one-way ANOVA with Sidak’s multiple comparisons test (Fig. 4A). *P* values comparing GC% and length between all transcripts with increased or decreased TE abundance transcripts were calculated with one-way ANOVA with Tukey’s multiple comparisons test (Fig. 5A, B). *P* values comparing GC% and length between host and RSV transcripts were calculated with one-way ANOVA with Sidak’s multiple comparisons test (Fig. 5C).

One-dimensional (Figs. 4A, 5A-C, Supplementary Figs. S3A, and S4B) and two-dimensional scatterplots (Supplementary Fig. S5A, D) displaying transcript’s GC% and length (nt) were generated using GraphPad Prism v.10.2.2.

Simple enrichment analysis (SEA) [37] against the RNA motif database (Ray2013 Homo sapiens) was used to uncover RNA-binding protein motifs within the 3’-UTRs of MANE and RSV sequences.

## Results

### Polysome occupancy is increased during RSV infection

While RSV activates the stress-induced eIF2α-phosphorylating kinase PKR (Supplementary Fig. S1A), consistent with previous studies [21, 22], the extent of downstream phosphorylation of eIF2α and consequent stress granule formation remains unclear [21, 22, 25–28]. We tested if eIF2α is phosphorylated during infection and found that only a small fraction is phosphorylated by western blot compared to cells treated with arsenite (NaAsO_2_) (Fig. 1A, B and Supplementary Fig. S1B). Similar results were observed at earlier RSV-infection timepoints (Supplementary Fig. S1C). To further validate that eIF2α remains unphosphorylated during infection, we also confirmed the absence of downstream stress granule formation in RSV-infected cells by indirect immunofluorescent staining using stress granule markers PABP and G3BP (Supplementary Figs. S1D, E) [38], which is in stark contrast with NaAsO_2_-treated cells (Supplementary Fig. S1F). Consistent with previous work [12], we observed that following RSV infection, cytoplasmic inclusion bodies were formed which function as sites of viral RNA transcription by the viral polymerase. These consist of viral proteins N, P, L and M2-1 and a selection of host proteins with functions in translation, including PABP (Supplementary Fig. S1D, zoom), but excluding *bona fide* stress granule marker G3BP (see Supplementary Fig. S1E, zoom) [12, 25]. Fig. 1.

To further test if lack of stress granule formation by RSV is caused by inhibition of eIF2α phosphorylation or by rapid dephosphorylation of eIF2α-P, we used NaAsO_2_ to activate another eIF2α-phosphorylating kinase, HRI [39] (as opposed to PKR which recognizes viral dsRNA). Any kinase activating eIF2α phosphorylation will reduce translation initiation and increase stress granule formation (Fig. 1C) [40]. We found that RSV-infected cells retained the ability to form stress granules after NaAsO_2_ treatment (Fig. 1D), consistent with previous work [18]. Next, the same experiment was performed with lower NaAsO_2_-concentrations to ensure that activation of the NaAsO_2_-activated stress signalling pathways was not overwhelming any potential RSV-induced inhibitory system. Consistent with the highest NaAsO_2_ concentration, we found no significant differences in stress granule formation between mock- and RSV-infected cells after NaAsO_2_ treatment (Fig. 1E), suggesting that infected cells are capable of stress granule formation.

Next, we performed polysome profiling to separate mRNAs according to the number of bound ribosomes. By fractionating lysates on sucrose gradients, we obtained separation between free RNA (not shown), 40S and 60S ribosomal subunits, 80S monosomes, and polysomes (Fig. 1F, G). Treatment with NaAsO_2_ results in a strong translational arrest seen by a large increase in the 80S peak and disappearance of polysomes (Fig. 1F), consistent with translation inhibition as shown previously [41, 42]. We expected to observe similar levels in polysomes between mock- and RSV-infected lysates, but interestingly we found that polysome levels are consistently increased as seen by an increase in the polysome/monosome ratio compared to mock-infected cells across all replicates in both HEp-2 and A549 cells (Fig. 1G and Supplementary Fig. S1G). The increase in polysomes is accompanied by a decrease in 80S monosomes, while 40S and 60S subunit levels remain similar (Fig. 1G and Supplementary Fig. S1G), indicating that 80S monosomes are being redistributed to the polysomes as opposed to an increased level of ribosome production. Overall, our findings demonstrate that during RSV infection, stress granules are absent, and polysome occupancy is increased.

### RSV infection induces distinct modes of host translation changes

To determine which transcripts are associated with heavy polysomes (4 or more ribosomes on one transcript) during infection, we isolated total and polysome-associated mRNA from mock- and RSV-infected cells and performed high-throughput sequencing after poly(A)-tail enrichment (as opposed to rRNA depletion) (Fig. 2A and Supplementary Table S1). Sequencing reads were mapped to the concatenated human and RSV genome. We confirmed RSV infection by western blot (Supplementary Fig. S2A) and RNA quality by agarose gel (Supplementary Fig. S2B). After mapping the reads, we observed high reproducibility between biological replicates (Supplementary Figs. S2C, D). Specific mapping of viral mRNAs (as opposed to the antigenome) was confirmed by the absence of reads in intergenic regions (Supplementary Fig. S2E). We observed slight increased levels of transcription readthrough at NS1-NS2 intergenic region, as described previously [43]. Fig. 2.

Next, the samples were processed to obtain DESeq2 normalized reads, hereafter referred to as normalized reads (Supplementary Tables S2 and S3, see methods for detailed description of DESeq2 normalization parameters [33]). Normalized reads were used by DESeq2 to calculate translation efficiencies (TE) by computing the ratio between polysomal and total mRNAs (Fig. 2A, Supplementary Table S4). TE is a measure of how well mRNAs become loaded with ribosomes. Since calculations were done with normalized reads, average TE numbers are expected to be around 1. While the focus of our study is on host transcripts, we noted that viral transcripts were more abundant in total RNA compared to heavy polysomal RNA at our time point of 24 h post-infection, leading to an apparent low TE (Supplementary Table S4). Many variables could contribute to this effect, such as the previously observed retention of nascent viral transcripts in inclusion bodies [10–12]; as such, viral RNAs could be abundant in total RNA extractions, yet relatively less accessible to polysomes than host mRNAs. Alternatively, the cell type or dependence on the course of the viral infection life cycle could play a role; for example, the TE of HIV transcripts change from 0.6% to 6% between 8 and 24 h post-infection [44].

Next, to determine how host transcripts are affected by RSV infection, we used DESeq to perform differential expression analysis between RSV- and mock-infected samples (see methods). In total mRNA, we observed that many host transcripts are significantly up- or downregulated during infection (Fig. 2B, light purple dots and triangles). We performed gene ontology (GO) of biological processes for the differentially expressed mRNAs and, as expected, found that genes that function in cytokine responses [45] and cell adhesion [46] were upregulated during RSV infection (Supplementary Fig. S2F, Supplementary Table S5). Consistently, we observed transcriptional upregulation of interferon-stimulated genes (ISGs) in RSV infected cells, indicating activation of the type I interferon antiviral response [47] (Fig. 2B, triangles). This includes the interferon-inducible antiviral proteins *OASL* [48] and *ISG15* (Fig. 2B, light purple labels indicating significantly changing transcripts) [49]. Other highlighted example transcripts remain unchanged (Fig. 2B, light grey and dark purple labels; discussed in more detail later).

Next, differential expression analysis of polysome-associated mRNAs likewise showed up- and down-regulation of many host transcripts during infection, including upregulation of ISGs that were noted above to similarly change in total RNA, including *OASL* [48] and *ISG15* [49] (Fig. 2C, light purple labels indicating significantly changing transcripts). Interestingly, GO biological processes are broadly consistent between total and polysome mRNA samples (Supplementary Fig. S2F, compare total and polysomal mRNA). This indicates that many transcripts that are transcriptionally upregulated recruit ribosomes and therefore also have a higher abundance in the polysomes. More specifically, ISG15 protein levels are known to be strongly upregulated during RSV infection [49], shown here to be caused by transcriptional upregulation of the *ISG15* transcript and translation of those new transcripts in polysomes. On the other hand, another ISG, *EIF2AK2/PKR*, is not transcriptionally upregulated (Fig. 2B, padj = 0.15; FC = 1.05) but has a significant shift in polysome abundance (padj < 0.05; FC = 1.45) (compare *ISG15* and *OASL* against *PKR*, Fig. 2F). Therefore, PKR protein levels mainly increase (see Supplementary Fig. S1A) due to enhanced translational efficiency of the mRNA while ISG15 increases mainly due to more abundant transcripts without a change in their translation efficiency.

To more broadly investigate how TE changes during infection, we performed differential expression analysis of the TE between RSV- and mock-infected cells (Supplementary Table S4). Interestingly, we found that more than 500 transcripts become significantly more efficient at ribosome recruitment (Fig. 2D, light purple dots). These include *THAP2*, *DHX9* and *SMC4* (padj < 0.05; FC > 1.5). DHX9 protein has been identified as both pro- and antiviral in different viruses [50] and *SMC4* has been shown to have an increased translation efficiency during HIV infections as well [44].

To further characterize how host transcripts are being enriched or depleted within polysomes during infection, we plotted differentially expressed transcripts (padj < 0.05) relative to mock-infected samples for polysomal mRNAs vs total mRNAs (Fig. 2E) [51]. We found that 40% of the changes in polysomal mRNA abundance were driven by changes in total mRNA, without changes in TE, seen by the distribution of datapoints along the diagonal (Fig. 2E, light grey). As expected, *ISG15* and *OASL* are found within this cohort (Fig. 2F). Interestingly, an additional 19% of transcripts followed this general trend where polysomal changes correlated with total RNA changes, but in a way that slightly amplified the effect from total RNA due to a significant change in TE (Fig. 2E, F, dark grey; *DHX9*). In addition, 15% of genes changed in their polysome association with no changes in total abundance, resulting in TE changes (Fig. 2E, F, light green; *PKR*, *THAP2* and *SMC4*). And lastly, we found that 26% of transcripts changed in total mRNA abundance but without corresponding changes in their association with polysomes, thus “buffering” (canceling out) the effect of transcription (Fig. 2E, F*,* dark green; *PITPNM1*). As a result, in these cases it is not expected that there would be changes in protein production. Overall, these data indicate that during RSV infection different types of translational regulation occur.

### Transcripts with low TE become more efficient at recruiting ribosomes than those with high TE during RSV infection

Given our observations above that some host mRNAs were selectively enriched or depleted in the heavy polysomes (independent of total RNA changes), we wanted to further investigate this effect of RSV-infection on TE. We plotted the TE of all protein-coding transcripts from RSV- against mock-infected samples after filtering low abundance transcripts (DESeq’s baseMean > 25) (Fig. 3A, Supplementary Table S6). Intriguingly, the data did not fall stochastically around the diagonal of the scatterplot but exhibited a clear pattern where the points generally fell above the diagonal for low TE transcripts and below for high TE transcripts. Fig. 3.

To better visualize this observation, we divided the plot based on high (> 2) and low (< 2) TE (Fig. 3B). We validated these cutoffs for high vs low TE, by comparing GC% and transcript length in our data. Generally, transcripts with a higher GC content [52] and shorter coding sequence (CDS) lengths [53, 54] are more efficiently translated (high TE). We plotted these features for the low and high TE datasets and confirm that the high TE transcripts (> 2) contain significantly shorter CDSs and higher GC-content (Supplementary Fig. S3A).

We then highlighted several transcripts of interest, including the previously described transcript *PITPNM1*, which is a high TE transcript with fourfold more mRNA in the polysomes compared to the input (TE equals 4.6 in mock-infected cells) (Fig. 3B, high TE, Supplementary Fig. S3B). During RSV infection, the TE of this transcript decreases to 3.3 (Supplementary Fig. S3B). While this is a substantial reduction in TE, this transcript remains efficient at ribosome recruitment. On the other hand, previously described significantly upregulated TE transcripts *DHX9*, *THAP2*, *SMC4* are examples of significantly upregulated low TE transcripts (Fig. 3B, low TE, Supplementary Fig. S3B).

Globally, the division between high and low TE transcripts further demonstrates that during RSV infection, normally highly translated host transcripts specifically appear to be less efficient at recruiting ribosomes (Fig. 3B, high TE), seen as a downwards curve from the diagonal when comparing the TE between mock- and RSV-infected cells. While transcripts with a high TE undergo a decrease in TE during viral infection, the opposite trend is observed for transcripts with a low TE (Fig. 3B, low TE). Overall, we observe more efficient recruitment of polysomes to many low TE transcripts (*n* = 6295) and a relative decrease in TE of high TE transcripts (*n* = 4023) during RSV infection. To visualize the distribution more quantitatively, we computed a histogram of the ratio of these values at each data point (Fig. 3C). In line with the density scatterplots, we observe a shift (right) of the distribution toward higher TE for low TE transcripts and a shift (left) of the distribution toward lower TE for high TE transcripts. It is important to note that the changes in TE observed for low TE transcripts are relatively small, such that high and low TE transcripts generally remain in their respective categories (Fig. 3A, purple boxes).

Overall, these findings demonstrate that host transcripts with a high TE that are normally highly capable of recruiting ribosomes become less efficient—relative to low TE transcripts—at getting translated during RSV infection (Fig. 3D). As this effect is relative, it is possible that it is driven by high TE transcripts reducing ribosome loading, low TE transcripts increasing ribosome loading, or a combination of both.

### VSV also induces a redistribution of ribosomes towards transcripts with low TE

Like RSV, vesicular stomatitis virus (VSV) transcribes monocistronic 5’-capped and polyadenylated transcripts [55]. In addition, transcript features such as GC% and length within the 5’-UTR, CDS and 3’-UTR are very similar between both viruses (Fig. 4A). Unlike RSV, previous studies demonstrated that VSV infection induces “host shutoff” resulting in a global reduction in host mRNA abundance [56–60] and through inhibition of host mRNA translation, without affecting viral translation [61, 62]. These characteristics make VSV an interesting virus to compare against RSV. Fig. 4.

To identify any ribosome redistribution trends within host mRNAs during VSV infection, we investigated a previously published high-throughput sequencing dataset of VSV infected total and polysomal mRNAs containing 2 or more ribosomes [34]. We used the same computational pipeline as for processing of RSV reads where we mapped sequencing reads to a concatenated human and VSV genome. Next, the samples were processed to obtain DESeq2 normalized reads, filtered to omit low abundance transcripts (DESeq’s baseMean > 25), and plotted the TE between mock- and VSV-infected cells, as done in Fig. 3A, B (Supplementary Table S7). We found that the changes in TE for VSV followed a similar trend compared to RSV (Fig. 4B). Similar to RSV, host transcripts with a high TE (> 1.5) (TE cut-off determined in Supplementary Fig. S4A to obtain similar number of represented transcripts) appear to be the least efficient at recruiting ribosomes (Fig. 4B, distribution towards the left in the histogram), compared to transcripts with a low TE (< 1.5) (Fig. 4B, distribution towards the right in the histogram).

As described previously, to confirm our cutoffs for TE, we plotted GC content [52] and CDS length [53, 54] and confirmed that the high TE transcripts (> 1.5) contain significantly shorter CDSs and higher GC-content (Supplementary Fig. S4B). Despite a more active host-shutoff mechanism in VSV, the similarity in TE changes suggest a shared underlying mechanism. Overall, these findings indicate that both RSV and VSV redistribute ribosomes from high TE host mRNA towards low TE mRNAs.

### Longer AU-rich transcripts are specifically enriched in heavy polysomes during RSV infection

To determine common features between mRNAs with significantly up- or downregulated TEs (see Fig. 2D, see Supplementary Table S4, padj < 0.05 and log2 FC ≤ − 0.58 or FC ≥ 0.58; *n* = 533 increased and *n* = 46 decreased) we determined the GC% and length for the most representative transcript using the Matched Annotation from NCBI and EMBL-EBI (MANE) transcriptome [36, 63]. Upon comparing GC% between the statistically significant cohorts, we found that RSV-induced translationally upregulated transcripts have a significantly lower GC% (AU-rich) and that translationally downregulated transcripts contain a significantly higher GC% (GC-rich) compared to all coding transcripts (Fig. 5A, full transcript, Supplementary Table S8). Next, to identify whether the entire transcript or a specific feature of the transcript (*i.e.* UTR or CDS) determines the GC-content of up- and downregulated transcripts, we determined the GC-content for 5’-UTR (excluding start codon), CDS (including start and stop codons) and 3’-UTR (excluding stop codon and poly(A) tail). The correlation between increased translation and lower GC% was most strongly linked to the coding sequence (CDS) and 3’-UTR, with slight changes in the 5’-UTR (Fig. 5A, Supplementary Table S8). To replicate these RNA-seq based observations, a random cohort of highly and lowly translated transcripts were selected (Supplementary Figs. S5A, B) and similar trends were observed by qRT-PCR (Supplementary Fig. S5C). Fig. 5.

In addition, more highly translated mRNAs during RSV infection appear to have a longer transcript length, again linked to the CDS and 3’-UTR (see Fig. 5B, Supplementary Table S8). To confirm that these two factors contribute independently, we showed that there is no correlation between GC% and transcript length (Supplementary Fig. S5D, R^2^ = 0.049). These data suggest that during RSV infection, longer AU-rich host transcripts are more efficient at recruiting ribosomes, while shorter GC-rich host transcripts are less efficient. This is consistent with the general trend we observed where transcripts with low TE, which are longer GC-poor transcripts, become more efficient at ribosome recruitment (see Figs. 3 and 4).

### Translationally upregulated host and viral mRNAs are both AU-rich

While most host transcripts have a GC-content of 35–60% (median 49%, lower 0.05 quartile 43% and upper 0.05 quartile 57%), RSV transcripts have a low GC-content (Fig. 5C, GC% range from 29 to 43%), which is reflected in each one’s 5’-UTR, CDS and 3’-UTR. Transcript lengths between virus and host are generally similar (Fig. 5C, length). These observations suggest that the translational landscape of host transcripts, being biased to favor transcripts that have low GC-content during infection, may reflect an underlying trend generated during RSV infection to enhance translation of viral transcripts. A similar trend towards increased translation of lengthy AU-rich transcripts has previously been described in VSV, which like RSV, encodes 5’-capped and polyadenylated transcripts [34], and causes the same relative enhanced ribosome recruitment for transcripts with low translation efficiency (see Fig. 4).

The UTRs of transcripts can contain many *cis*-acting regulatory elements to regulate translation. These include stem-loops, IRESs and upstream open reading frames (ORFs) in the 5’-UTR, as well as sequences that can be recognized by regulatory RNA-binding proteins, polyadenylation elements and the poly(A) tail in the 3’-UTR [64]. Since RSV 5’-UTRs are very short (Fig. 5C) and the 5’-UTR GC-content and length of differentially translated host mRNAs during RSV infection is similar (Fig. 5A, B), the potential for regulatory elements within these sequences is relatively low. In contrast, host transcripts with 3’-UTRs that were AU-rich tended to be translated better (Fig. 5A). Therefore, we focussed on elements found within the 3’-UTR of differentially translated host and viral mRNAs.

First, we compared the poly(A) tail length between differentially expressed and translated mRNAs. We used the previously published dataset which determined average poly(A) tail length in HeLa cells [65], but found no statistically significant differences between translationally up- or downregulated transcripts (data not shown), which is consistent with previous findings where poly(A) tails length did not correlate with TE [65]. Next, many RNA-binding proteins are known to specifically recognize and conserved sequence elements [66] and a large number of RNA-binding proteins are known to affect translation of specific transcripts through regulatory elements found within the 3’-UTR [67, 68]. We used simple enrichment analysis (SEA) [37] to determine the occurrence of previously described RNA-binding protein motifs within the 3’-UTRs of translationally upregulated transcripts in RSV infected cells. We identified six major sequence motifs within this group of mRNAs (Supplementary Fig. S6) and compared these RNA-binding protein motifs against motifs identified within 3’-UTRs of viral transcripts and found multiple comparable groups (Supplementary Fig. S6). This data indicates that 3’-UTR binding host proteins could regulate a shift in AU-rich translation during viral infection.

## Discussion

### RSV infection maintains translation and redistributes 80S monosomes into the translating pool of ribosomes

**Fig. 6:**
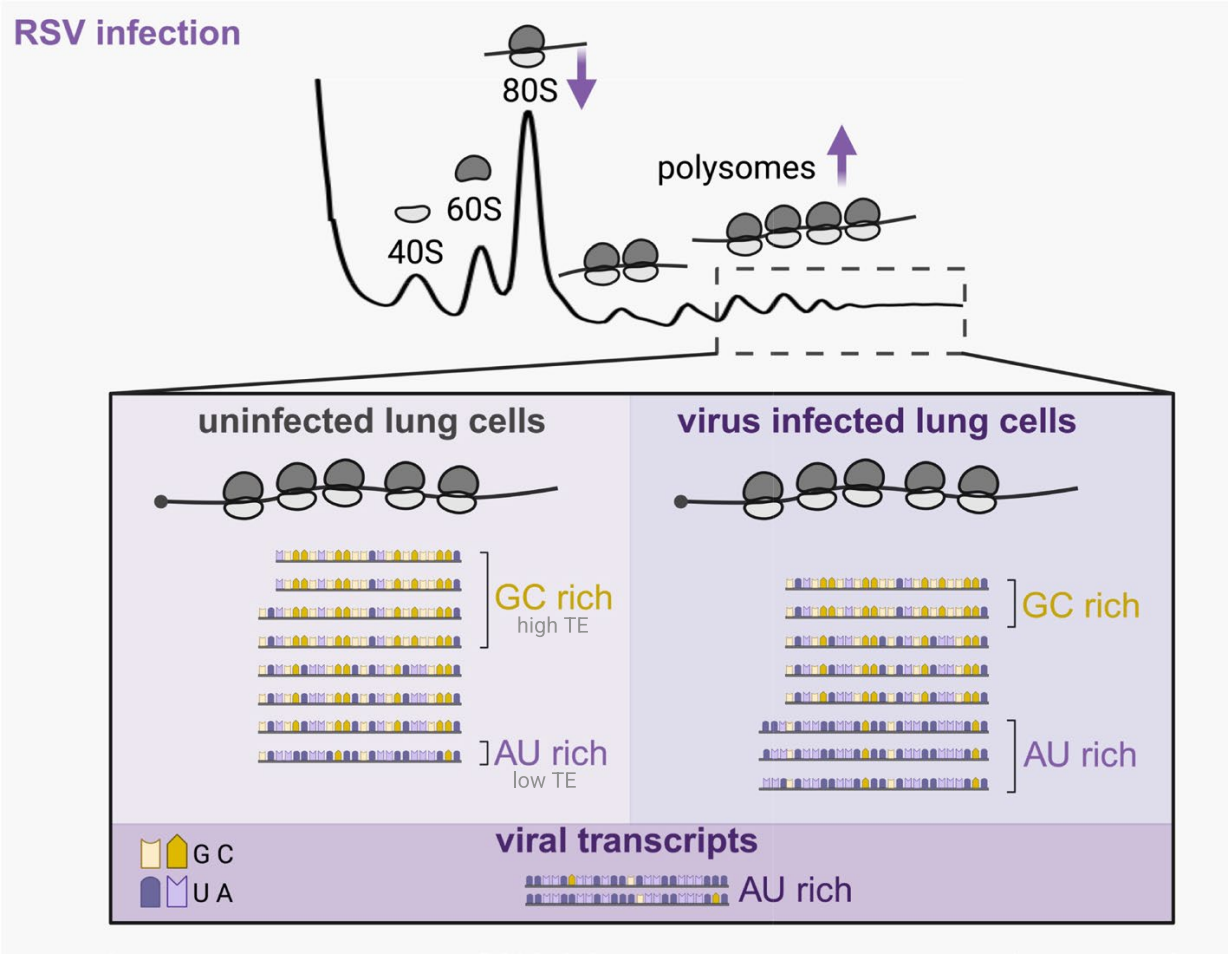
Model summarizing translational changes during RSV infection. RSV infection of HEp2 cells leads to a redistribution of ribosomes towards the polysomes. The 80S monosome peak is consistently smaller, while the polysome peaks are larger. RNA sequencing of total RNA and heavy polysomes revealed that AU-rich low TE transcripts are more efficient at recruiting ribosomes during RSV infection. This is seen as an enhanced translation efficiency (TE) for these transcripts compared to mock-infected cells. On the other hand, GC-rich high TE transcripts have a decreased translation efficiency. Overall, ribosomes are redistributed during RSV infection resulting in a relative increase of translation efficiency of normally AU-rich low TE transcripts which resemble viral transcripts

We showed that translation initiation was not inhibited through polysome profiling and instead found that polysome peaks are increased during RSV infection (Fig. 6). This was accompanied by a decrease in 80S monosomes, indicating that monosomes are being redistributed to the polysomes. While we cannot exclude an increased total number of ribosomes in the cell, the ribosome redistribution from monosomes to polysomes indicates that more ribosomes are associated with mRNAs during RSV infection.

While polysome profiling is a powerful method to obtain a global overview of the distribution of 80S ribosomes compared to polysomes, it is important to note that not all transcripts found within polysomal fractions are undergoing active translation. More specifically, ribosome pausing occurs relatively frequently during translation [69]. Rare codons, caused by low availability of matching tRNAs, are known to cause elongating ribosome to pause [70, 71]. Ribosomal pausing could be utilized by RSV to promote co-translational folding of viral proteins or to enhance endoplasmic reticulum (ER)-targeting of viral membrane proteins RSV-G, -F and -SH. Additionally, viruses often employ ribosomal pausing through a slippery sequence to induce programmed ribosomal frameshifts to enhance their coding capacity [72]. However, when ribosomes undergo prolonged pausing, the potential for ribosome collisions exists which leads to formation of ribosome complexes containing two (disomes), three (trisomes) or more ribosomes [73]. While ribosome collisions are eventually resolved by several surveillance pathways [73], this could result in larger polysomes. More specifically, treatment with intermediate concentrations of translation elongation inhibitors (such as anisomycin), leads to increased ribosome collision and has been found to decrease 80S monosomes and increase polysomes [74], similar to our polysome traces comparing mock- and RSV-infected cells.

The number of ribosomes found within polysomes is determined by a combination of translation initiation and elongation rates, where faster translation initiation and slower translation elongation both enhance polysome formation [75, 76]. In addition, larger polysome peaks can also be induced by increased polysome-association by transcripts with longer CDSs which can accommodate more ribosomes. It has indeed been shown that the number of ribosomes associated with a transcript correlates with the CDS length [77] and that transcripts with short ORFs (< 500 nt) are typically found more frequently as 80S monosomes as opposed to polysomes [75]. We found that during RSV infection transcripts with longer CDSs are indeed specifically enriched in polysomes which could contribute to the observed increased polysome peaks.

### Low polysome occupancy transcripts are longer and AU-rich and become more efficient at ribosome recruitment during infection

While previous work has shown that viral proteins alter host transcription through direct chromatin interactions [78, 79], limited information on translational changes have been described to date. We found that during RSV infection, ribosomes get redistributed from GC-rich transcripts that are normally efficient at ribosome recruitment to AU-rich transcripts that are less efficient (Fig. 6). This global shift in higher ribosome recruitment on AU-rich transcripts could benefit translation of the AU-rich viral transcripts. In particular, since viral transcripts are produced as mature transcripts in cytoplasmic inclusion bodies by the viral polymerase [12], these mRNAs do not undergo the same maturation pathway as host mRNAs, including splicing, RNA-binding protein interactions and nuclear export. Importantly, it has been shown that intron splicing generally enhances gene expression [80]. Another potential workaround to successfully compete with host transcripts for ribosomes is to directly recruit ribosomes to inclusion bodies for translation, as described in [18]. On the other hand, the host could also benefit from a change in the translational landscape. It remains to be determined whether certain antiviral host transcripts are translationally upregulated during RSV infection and as a result reduce viral infection.

A possible cause for the global redistribution of ribosomes from GC-rich high TE to AU-rich low TE transcripts is mature tRNA level availability. More specifically, decreased availability of specific mature tRNAs could decrease translation for a selection of transcripts [81] since translation elongation slowdowns decrease translation initiation rates [82–85]. A recent study however has found that while mature tRNA levels are different during differentiation, the tRNA anticodon pool remains the same which maintains the decoding speed of elongating ribosomes [86]. Similarly, it has been shown that while large differences exist in isodecoder expression in different tissues, the anticodon pool remains similar [87]. It remains to be determined if these rules are also valid during viral infections and if RSV induces changes in mature tRNA levels that could cause these global ribosome redistributions from GC-rich to AU-rich transcripts.

## Conclusion

An important question for viruses that do not induce “host shutoff” is how they successfully compete with host transcripts for ribosomes required for translation of their viral transcripts. We found that RSV maintains global translation and that ribosomes are redistributed from host transcripts that are normally highly efficient at ribosome recruitment to host transcripts that are less efficient. We also describe that RSV transcripts consist of AU-rich sequences. Interestingly, host transcripts with significantly increased translation efficiency (TE) were found to be longer and AU-rich, indicating that the translational landscape of host transcripts may reflect an underlying trend that is created by RSV to enhance translation of viral transcripts.

## Supplementary Information

Supplementary Material 1: Supplementary Fig. S1. Related to Fig. 1. RSV infection does not induce stress granule formation in HEp2 and A549 cells. Western blot demonstrating PKR upregulation during RSV infection. GAPDH serves as a loading control. RSV infection only induces low levels of eIF2α phosphorylation. Western blot demonstrating lack of strong eIF2α phosphorylation during RSV infection by comparing eIF2α-P and total eIF2α levels between mock- and RSV-infectedand untreated and NaAsO_2_-treatedin A549 cells. RSV infection was confirmed by immunoblotting with a polyclonal anti-RSV antibody. β-actin serves as a loading control. Relative quantification against control samples. *P* values were calculated with one-way ANOVA with Sidak’s multiple comparisons test.Western blot comparing eIF2α-P and total eIF2α levels between mock- and RSV-infectedat different time points. Viral proteins were detected using a polyclonal anti-RSV antibody. *P* values were calculated with one-way ANOVA with Sidak’s multiple comparisons test.RSV infection does not induce stress granule formation seen by indirect immunofluorescent staining of mock- and RSV-infected cellsvia stress granule markers PABPand G3BP. RSV proteins were detected using a polyclonal anti-RSV antibodyand nuclei were stained using DAPI. The white box corresponds to 10 μm and is enlarged in the zoom panel to visualize viral inclusion bodies where nascent viral transcripts are transcribed. Inclusion bodies are known to contain PABP.Stress granule formation seen by indirect immunofluorescent staining of arsenite-treated cellsdetecting stress granule markers PABP and G3BP. Nuclei were stained using DAPI.Polysome profiles of sucrose gradient fractionated mock- and RSV-infected A549 cells. AUC quantification between polysomes and monosomesare plotted to estimate translation levels. AUC quantification between free RNA fractionand 40S, 60S and 80S are plotted to determine changes in free monosomes and 80S subunits. *P* values were calculated with an unpaired t-test for polysome vs monosome comparisons and a two-way ANOVA with Sidak’s multiple comparisons test. AUC: area under the curve.

Supplementary Material 2: Supplementary Fig. S2. Related to Fig. 2. Quality control and GO analysis of RNA-seq samples.Western blot of total cytoplasmic protein obtained from samples used for high-throughput sequencing immunoblotted with polyclonal antibodyanti-RSV and monoclonal antibodies anti-RSV-N, anti-RSV-P and anti-RSV-M2-1 to confirm viral infection and loading control GAPDH.Agarose gel to determine RNA quality of purified RNA samplesselection). Note the absence of tRNAs in the polysomal RNA, unlike total RNA where free tRNA is abundant.Multidimensional scalingto determine similarity between RNAseq replicates. Diversity between samples is delineated by RNA typeand infection status.Heatmap demonstrating reproducibility between biological replicates. Color gradient shown on the heatmap corresponds to the Euclidian distance which was calculated for gene expression matrixes and compared between samples. Biological replicates are similar in distance and cluster together.IGV snapshot of the RSV genome of total RSV-infected samples. Individual viral mRNAs are annotated below each gene. Read coverage is specific to genomic regionsand absent in intergenic regions indicating specific sequencing of viral mRNAs as opposed to viral genome contamination. Some transcription readthrough exists between NS1 and NS2 as described earlier [43].Gene ontologyanalysis of biological processes for upregulated total mRNA, polysomal mRNA and TE during RSV infection. A large overlap exists between total and polysomal mRNAs indicating that transcripts that are increasing in abundance are also increased in the polysomes. On the other hand, the GO terms for TE are completely different.

Supplementary Material 3: Supplementary Fig. S3. Related to Fig. 3. TE quality control data for RSV data.Distribution of the GC% and CDS length of host protein-coding transcripts divided between high TEand low TE. P values were calculated with an unpaired t test. Highly translated mRNAs are shorter and GC-rich.Bar plots summarizing normalized read counts for transcripts with a significant TE. *PITPNM1* is a high TE transcriptand *DHX9*, *THAP2* and *SMC4* are low TE transcripts.

Supplementary Material 4: Supplementary Fig. S4. Related to Fig. 4. TE quality control data for VSV data.Cumulative histograms of TE ratiosto determine high vs. low TE cut-offs. Data to determine TE cut-off was derived from uninfected samples for both the RSV datasetand VSV dataset. The RSV dataset was divided between high and low TE transcripts by setting the cut-off value at 2. This approximately separates the top 34% most highly translating transcriptsfrom the other 66% transcripts with low TE. On the other hand, setting a TE cut-off value of 2 for the VSV dataset would result in a division of 89%vs. 12%. Since this would likely result in a non-representative dataset, we set the TE cut-off for the VSV dataset at 1.5 which divides the reads 72% - 28%.Distribution of the GC% and CDS length of host protein-coding transcripts divided between high TEand low TEfrom previously published dataset from Neidermyer *et al*. 2019. P values were calculated with an unpaired t test. Highly translated mRNAs are shorter and GC-rich.

Supplementary Material 5: Supplementary Fig. S5. Related to Fig. 5. Total and polysome-associated differentially expressed protein-coding transcripts.GC% and transcript length from the random cohort of highly and lowly translated transcripts confirmed in B and C.A selection of transcripts from the RNAseq dataset in B shown to be consistent by qRT-PCR in C. Translation efficiencyfor RSV/mock fold enrichment was calculated by the ratios of ΔΔCt normalized against 5.8S rRNA.Scatterplots demonstrating no correlation between GC% and transcript length.

Supplementary Material 6: Supplementary Fig. S6. Related to Fig. 5. Similarities between 3’-UTR sequence motifs between viral and translationally upregulated mRNAs. Simple enrichment analysisof motifs found within the 3’-UTR of statistically significantly translationally upregulatedprotein-coding transcriptscompared to the 3’-UTR of viral transcripts. In several cases, the same motif was found in both host and viral transcripts.

Supplementary Material 7: Supplementary Table S1. RNA-seq raw counts from this study, related to Fig. 2. Columns A-E contain gene information. Columns F-K mock-infected and columns L-Q RSV-infected raw counts. Total indicates total RNA and pol indicates polysome-associated RNA. Supplementary Table S2. DESeq2 normalized counts and DESeq2 differential expression for total RNA from this study, related to Fig. 2B. Columns A-E contain gene information. Columns F-K DESeq2 contain outputs for RSV/mock comparisons for total RNA. Columns L-Q contain normalized total counts. Supplementary Table S3. DESeq2 normalized counts and DESeq2 differential expression for polysome-associated RNA from this study, related to Fig. 2C. Columns A-E contain gene information. Columns F-K DESeq2 contain outputs for RSV/mock comparisons for polysome-associated RNA. Columns L-Q contain normalized polysome-associated counts. Supplementary Table S4. DESeq2 normalized counts and DESeq2 differential expression for translation efficiencies from this study, related to Fig. 2D. Columns A-E contain gene information. Columns F-K DESeq2 outputs for translation efficiency between RSV- and mock- infected cells. Columns L-Q normalized mock-infected counts and columns R-W normalized RSV-infected counts. Columns X-Y shows individual manually calculated TE for mock- and RSV-infected cells. Supplementary Table S5: GO analysis lists of differentially upregulated transcripts. Gene names of significantly upregulated total mRNAs, polysomal mRNAs and TE used for input files for GO analysis. TE: translation efficiency. Supplementary Table S6. DESeq2 differential expression for the translation efficiency data for mock and RSV infected samples from this study, related to Fig. 3. Columns A-D contain gene information. Columns E-I DESeq2 outputs for translation efficiency for mock-infected samples. Columns J-N DESeq2 outputs for translation efficiency for RSV-infected samples. Supplementary Table S7. DESeq2 differential expression for the translation efficiencydata for mock and VSV samples from [34], related to Fig. 4. Columns A-D contain gene information. Columns E-I DESeq2 outputs for translation efficiency for mock-infected samples. Columns J-N DESeq2 outputs for translation efficiency for VSV-infected samples. Supplementary Table S8. GC% and length data of MANE selected transcripts, related to Figs. 4 and 5. GC% and length for cDNA sequences, 5’-UTR, CDS and 3’-UTR. Transcripts were selected from the Matched Annotation from the NCBI and EMBL-EBI to obtain information for representative transcripts within the human transcriptome. Supplementary Table S9. Oligonucleotides used in this study. Related to Methods. List of oligos used for qRT-PCR.

## Data Availability

High-throughput RNA sequencing has been deposited to the Gene Expression Omnibus (GEO) under the accession number GSE268742 (https://www.ncbi.nlm.nih.gov/geo/query/acc.cgi?acc=GSE268742).

## Abbreviations

eIF2α: Eukaryotic initiator factor 2α
BSA: Bovine serum albumin
ER: Endoplasmic reticulum
FC: Fold change
MANE: Matched annotation from the NCBI and EMBL-EBI
MDS: Multidimensional scaling
NaAsO_2_: Sodium arsenite
ORF: Open-reading frame
RSV: Respiratory syncytial virus
SDS: Sodium dodecyl sulfate
SEA: Simple enrichment analysis
TBS-T: Tris-buffered saline Tween-20
TCID_50_: Tissue culture infectious dose-50
TE: Translation efficiency
VSV: Vesicular stomatitis virus
