# Supplementary figures and images for: Respiratory syncytial virus (RSV) enhances translation of virus-resembling AU-rich host transcripts
Source: Virol J. 2025 Jul 15;22:244. doi: 10.1186/s12985-025-02838-z (PMC12265200; doi:10.1186/s12985-025-02838-z)

Figure S1

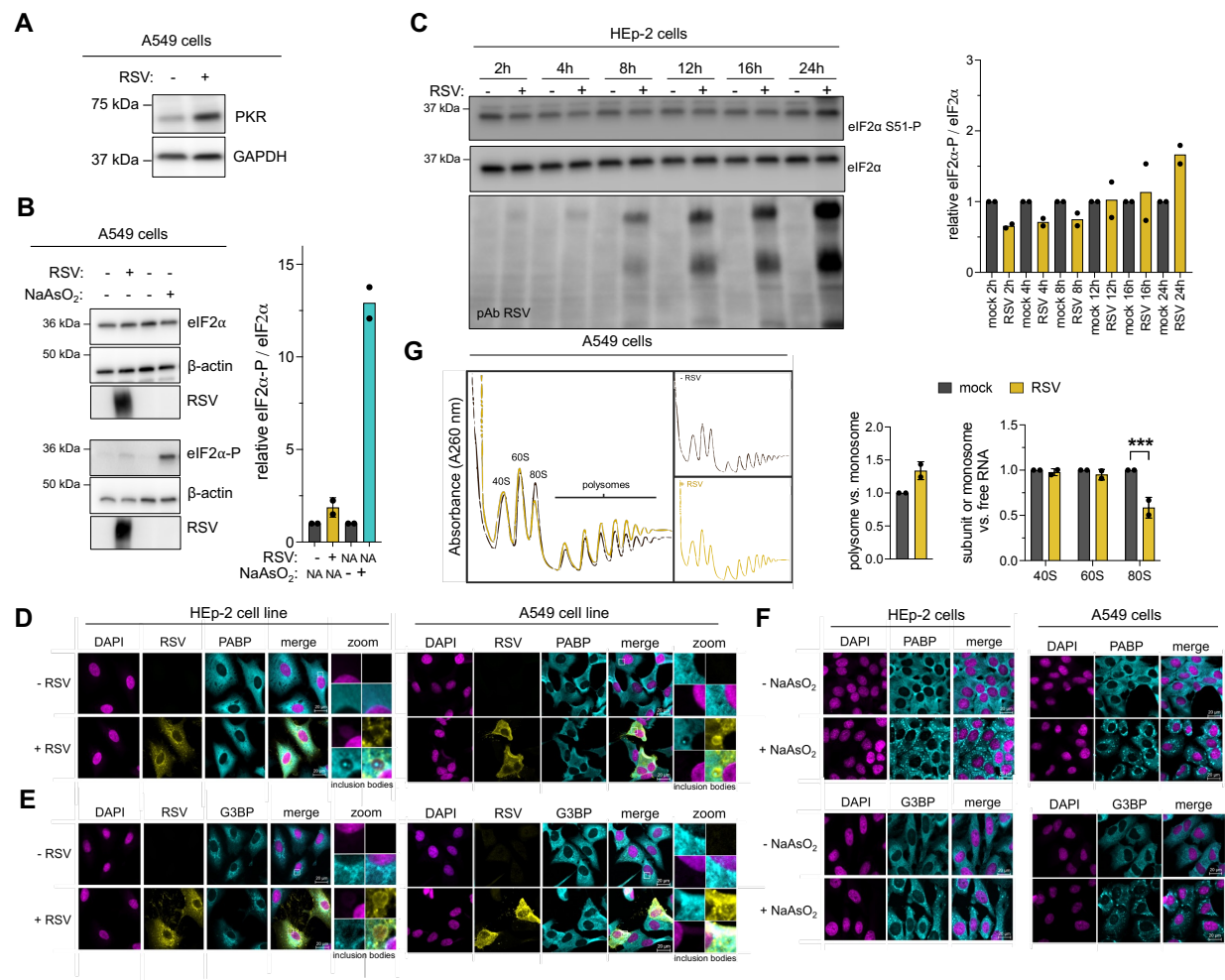

Supplement: Supplementary file 1 — Supplementary Material 1: Supplementary Fig. S1. Related to Fig. 1. RSV infection does not induce stress granule formation in HEp2 and A549 cells. Western blot demonstrating PKR upregulation during RSV infection. GAPDH serves as a loading control. RSV infection only induces low levels of eIF2α phosphorylation. Western blot demonstrating lack of strong eIF2α phosphorylation during RSV infection by comparing eIF2α-P and total eIF2α levels between mock- and RSV-infectedand untreated and NaAsO2-treatedin A549 cells. RSV infection was confirmed by immunoblotting with a polyclonal anti-RSV antibody. β-actin serves as a loading control. Relative quantification against control samples. P values were calculated with one-way ANOVA with Sidak’s multiple comparisons test.Western blot comparing eIF2α-P and total eIF2α levels between mock- and RSV-infectedat different time points. Viral proteins were detected using a polyclonal anti-RSV antibody. P values were calculated with one-way ANOVA with Sidak’s multiple comparisons test.RSV infection does not induce stress granule formation seen by indirect immunofluorescent staining of mock- and RSV-infected cellsvia stress granule markers PABPand G3BP. RSV proteins were detected using a polyclonal anti-RSV antibodyand nuclei were stained using DAPI. The white box corresponds to 10 μm and is enlarged in the zoom panel to visualize viral inclusion bodies where nascent viral transcripts are transcribed. Inclusion bodies are known to contain PABP.Stress granule formation seen by indirect immunofluorescent staining of arsenite-treated cellsdetecting stress granule markers PABP and G3BP. Nuclei were stained using DAPI.Polysome profiles of sucrose gradient fractionated mock- and RSV-infected A549 cells. AUC quantification between polysomes and monosomesare plotted to estimate translation levels. AUC quantification between free RNA fractionand 40S, 60S and 80S are plotted to determine changes in free monosomes and 80S subunits. P values were ca [file 12985_2025_2838_MOESM1_ESM.pdf]

Figure S2

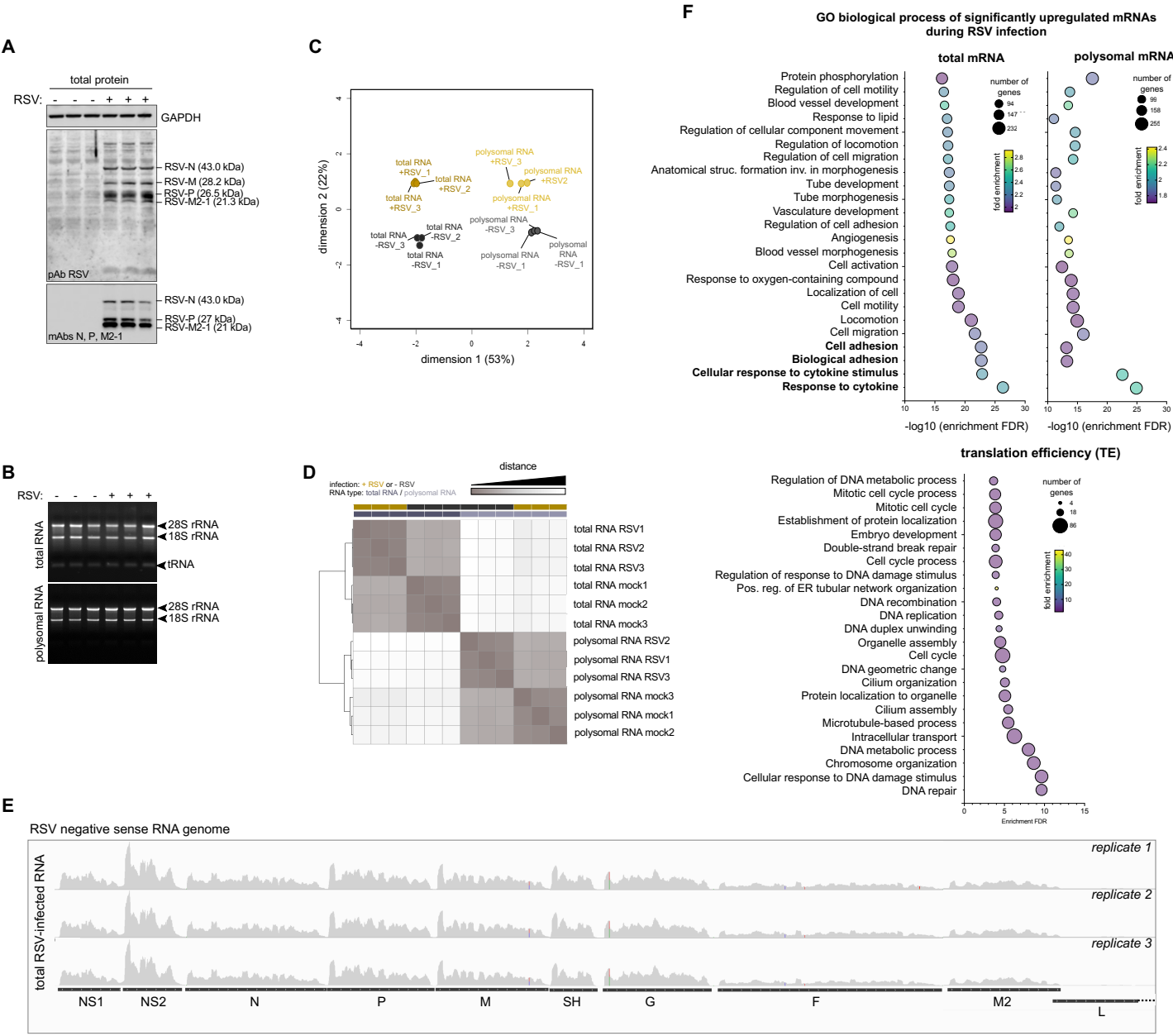

Supplement: Supplementary file 2 — Supplementary Material 2: Supplementary Fig. S2. Related to Fig. 2. Quality control and GO analysis of RNA-seq samples.Western blot of total cytoplasmic protein obtained from samples used for high-throughput sequencing immunoblotted with polyclonal antibodyanti-RSV and monoclonal antibodies anti-RSV-N, anti-RSV-P and anti-RSV-M2-1 to confirm viral infection and loading control GAPDH.Agarose gel to determine RNA quality of purified RNA samplesselection). Note the absence of tRNAs in the polysomal RNA, unlike total RNA where free tRNA is abundant.Multidimensional scalingto determine similarity between RNAseq replicates. Diversity between samples is delineated by RNA typeand infection status.Heatmap demonstrating reproducibility between biological replicates. Color gradient shown on the heatmap corresponds to the Euclidian distance which was calculated for gene expression matrixes and compared between samples. Biological replicates are similar in distance and cluster together.IGV snapshot of the RSV genome of total RSV-infected samples. Individual viral mRNAs are annotated below each gene. Read coverage is specific to genomic regionsand absent in intergenic regions indicating specific sequencing of viral mRNAs as opposed to viral genome contamination. Some transcription readthrough exists between NS1 and NS2 as described earlier [43].Gene ontologyanalysis of biological processes for upregulated total mRNA, polysomal mRNA and TE during RSV infection. A large overlap exists between total and polysomal mRNAs indicating that transcripts that are increasing in abundance are also increased in the polysomes. On the other hand, the GO terms for TE are completely different. [file 12985_2025_2838_MOESM2_ESM.pdf]

Figure S3

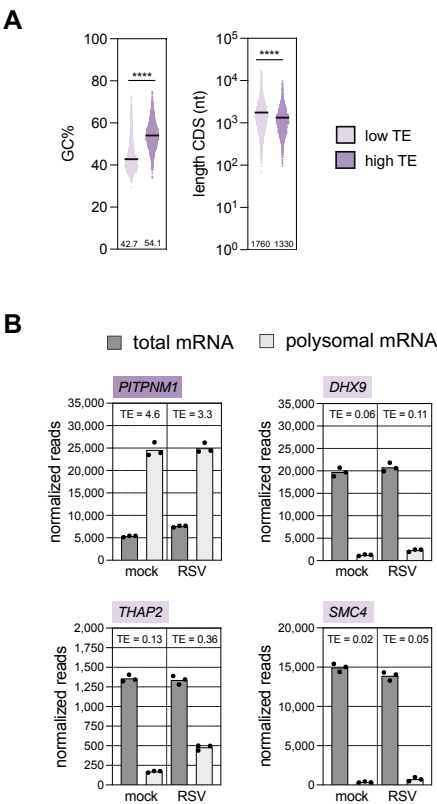

Supplement: Supplementary file 3 — Supplementary Material 3: Supplementary Fig. S3. Related to Fig. 3. TE quality control data for RSV data.Distribution of the GC% and CDS length of host protein-coding transcripts divided between high TEand low TE. P values were calculated with an unpaired t test. Highly translated mRNAs are shorter and GC-rich.Bar plots summarizing normalized read counts for transcripts with a significant TE. PITPNM1 is a high TE transcriptand DHX9, THAP2 and SMC4 are low TE transcripts. [file 12985_2025_2838_MOESM3_ESM.pdf]

Figure S4

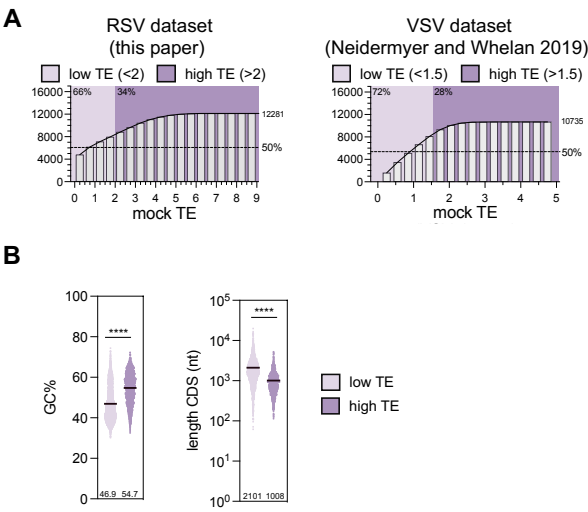

Supplement: Supplementary file 4 — Supplementary Material 4: Supplementary Fig. S4. Related to Fig. 4. TE quality control data for VSV data.Cumulative histograms of TE ratiosto determine high vs. low TE cut-offs. Data to determine TE cut-off was derived from uninfected samples for both the RSV datasetand VSV dataset. The RSV dataset was divided between high and low TE transcripts by setting the cut-off value at 2. This approximately separates the top 34% most highly translating transcriptsfrom the other 66% transcripts with low TE. On the other hand, setting a TE cut-off value of 2 for the VSV dataset would result in a division of 89%vs. 12%. Since this would likely result in a non-representative dataset, we set the TE cut-off for the VSV dataset at 1.5 which divides the reads 72% - 28%.Distribution of the GC% and CDS length of host protein-coding transcripts divided between high TEand low TEfrom previously published dataset from Neidermyer et al. 2019. P values were calculated with an unpaired t test. Highly translated mRNAs are shorter and GC-rich. [file 12985_2025_2838_MOESM4_ESM.pdf]

Figure S5

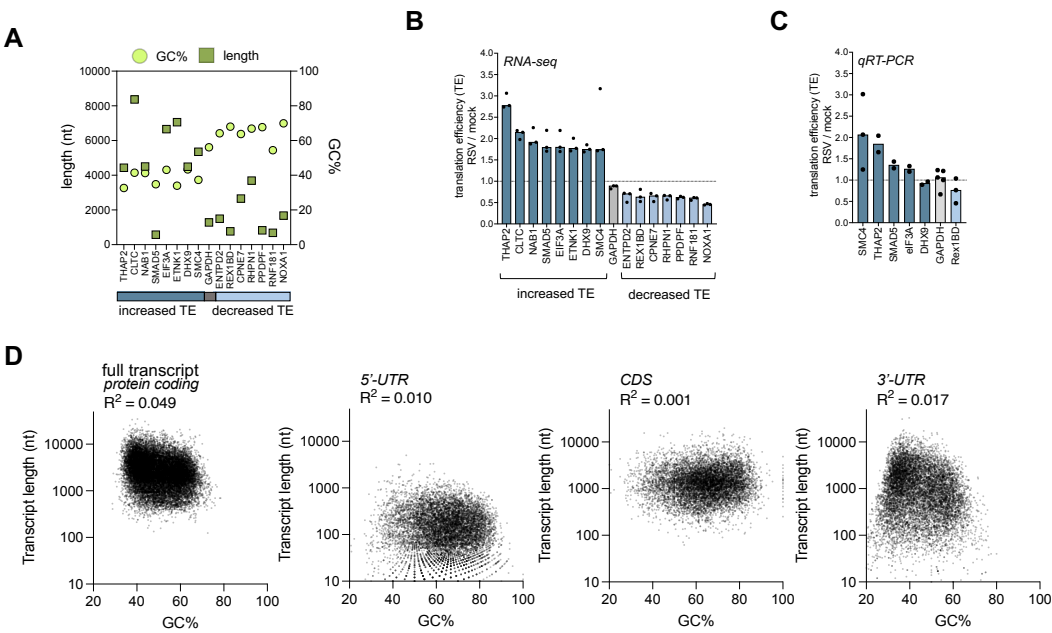

Supplement: Supplementary file 5 — Supplementary Material 5: Supplementary Fig. S5. Related to Fig. 5. Total and polysome-associated differentially expressed protein-coding transcripts.GC% and transcript length from the random cohort of highly and lowly translated transcripts confirmed in B and C.A selection of transcripts from the RNAseq dataset in B shown to be consistent by qRT-PCR in C. Translation efficiencyfor RSV/mock fold enrichment was calculated by the ratios of ΔΔCt normalized against 5.8S rRNA.Scatterplots demonstrating no correlation between GC% and transcript length. [file 12985_2025_2838_MOESM5_ESM.pdf]
