# Supplementary material for: Respiratory syncytial virus (RSV) enhances translation of virus-resembling AU-rich host transcripts
Source: Virol J. 2025 Jul 15;22:244. doi: 10.1186/s12985-025-02838-z (PMC12265200; doi:10.1186/s12985-025-02838-z)
Supplement: Supplementary file 6 — Supplementary Material 6: Supplementary Fig. S6. Related to Fig. 5. Similarities between 3’-UTR sequence motifs between viral and translationally upregulated mRNAs. Simple enrichment analysisof motifs found within the 3’-UTR of statistically significantly translationally upregulatedprotein-coding transcriptscompared to the 3’-UTR of viral transcripts. In several cases, the same motif was found in both host and viral transcripts. [file 12985_2025_2838_MOESM6_ESM.pdf]

Figure S6

Number of targets:	SEA motif enrichment 3'-UTR increased translation 313 genes total	Enrichment ratio:	RNA-binding protein:	Number of targets:	SEA motif enrichment 3'-UTR RSV transcripts 10 genes total	Enrichment ratio:	RNA-binding protein:
22	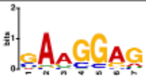	1.92	RBM5	4	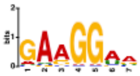	5.00	RBM5
19	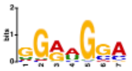	3.3	SRSF9				
13	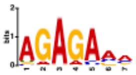	14	SRSF10				
45	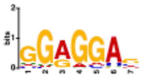	1.48	SRSF1				
132	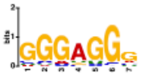	1.39	HNRNPH2	5	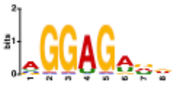	3.00	SRSF2
48	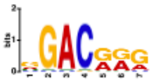	1.48	FXR2	6	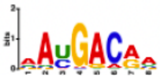	3.5	FXR1
				6	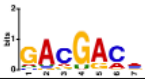	3.50	RBM45
96	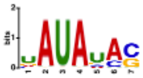	1.26	RBMS1	4	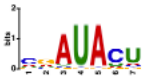	5.00	TUT1
144	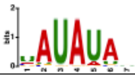	1.32	RBMS3				
35	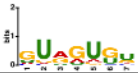	1.57	SFPQ		*no matching motif*		
126	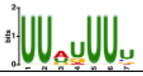	1.22	HuR		*no matching motif*		
90	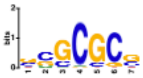	1.42	PPRC1		*no matching motif*		
	no matching motif			7	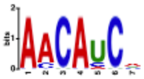	2.67	YBX1
